# Amino Acids in Cerebrospinal Fluid of Patients with Aneurysmal Subarachnoid Haemorrhage: An Observational Study

**DOI:** 10.3389/fneur.2017.00438

**Published:** 2017-08-28

**Authors:** Bartosz Sokół, Bartosz Urbaniak, Norbert Wąsik, Szymon Plewa, Agnieszka Klupczyńska, Roman Jankowski, Barbara Więckowska, Robert Juszkat, Zenon Kokot

**Affiliations:** ^1^Department of Neurosurgery, Poznan University of Medical Sciences, Poznan, Poland; ^2^Faculty of Pharmacy, Department of Inorganic and Analytical Chemistry, Poznan University of Medical Sciences, Poznan, Poland; ^3^Department of Computer Science and Statistics, Poznan University of Medical Sciences, Poznan, Poland; ^4^Department of General and Interventional Radiology, Poznan University of Medical Sciences, Poznan, Poland

**Keywords:** subarachnoid haemorrhage, amino acids, early brain injury, delayed cerebral ischaemia, biomarkers

## Abstract

**Background:**

The authors are aware of only one article investigating amino acid concentrations in cerebrospinal fluid (CSF) in patients with ruptured intracranial aneurysms, and this was published 31 years ago. Since then, both management of subarachnoid haemorrhage (SAH) and amino acid assay techniques have seen radical alterations, yet the pathophysiology of SAH remains unclear.

**Objective:**

To analyse the pattern of concentrations of amino acids and related compounds in patients with different outcomes following aneurysmal SAH.

**Methods:**

49 CSF samples were collected from 23 patients on days 0–3, 5, and 10 post-SAH. Concentrations of 33 amino acids and related compounds were assayed by liquid chromatography tandem mass spectrometry in patients with good [Glasgow Outcome Scale (GOS) 1–3] and poor (GOS 4–5) outcome.

**Results:**

Of the 33 compounds assayed, only hydroxyproline and 3-aminoisobutyric acid appeared not to increase significantly following SAH. In poor outcome patients, we found significantly higher concentrations of aspartic acid (*p* = 0.038), glutamic acid (*p* = 0.038), and seven other compounds on days 0–3 post-SAH; glutamic acid (*p* = 0.041) on day 5 post-SAH, and 2-aminoadipic acid (*p* = 0.033) on day 10 post-SAH. The most significant correlation with GOS at 3 months was found for aminoadipic acid on day 10 post-SAH (cc = −0.81).

**Conclusion:**

Aneurysmal rupture leads to a generalised increase of amino acids and related compounds in CSF. The patterns differ between good and poor outcome cases. Increased excitatory amino acids are strongly indicative of poor outcome.

## Introduction

Subarachnoid hemorrhage (SAH) due to ruptured intracranial aneurysms is a life-threatening condition with an annual incidence of 2–22.5/100,000 population per annum. The average annual attack rate per 100,000 population for men and women aged 25–64 years in Poland is 10.9 and 9.1, respectively, and the 28 day fatality rates 39 and 44% (1, 2). Cumulative morbidity and mortality following SAH remain high, despite the considerable efforts of neuroclinicians worldwide (3, 4). Traditionally, rebleeding and cerebral vasospasm have been regarded as the main causes of poor outcome in these cases (5). Although cerebral vasospasm has been extensively studied, and subjected to numerous drug trials during the past several decades, the outcome appears not to have been improved by its reversal (2, 6, 7). Several recent studies indicate that early brain injury (EBI), which occurs during the 24–72 h following aneurysmal rupture, makes a significant contribution to patient outcome, and may be responsible for the detrimental effects seen in patients after SAH (3, 8–10). EBI was first reported by Kusaka et al. (11). Since then, the knowledge of mechanisms involved in EBI significantly progressed, but it still warrants further investigation (12–20). During EBI, the central nervous system (CNS) suffers from “primary” insults (involving acute changes of intracranial pressure, cerebral perfusion pressure, and cerebral blood flow with vascular constriction and obstruction of the microcirculation), and “secondary” ischaemic processes (including anaerobic cellular respiration, energy depletion, impaired protein synthesis, excitotoxicity, free radical attack, neuronal stress, and DNA damage, leading to apoptosis and necrosis) (16). Many of these processes may potentially be initiated, mediated, or terminated by amino acids and related compounds. Von Holst and Hagenfeldt in 1985 appear to be the only group to have demonstrated increased levels of amino acids in cerebrospinal fluid (CSF) after SAH, and proposed mechanisms leading to it (21). Since then, no new studies analysing CSF amino acids involved in SAH has been published. Our present aim is to look into the role of amino acids and related compounds potentially involved in the process of brain injury following SAH. Determination of amino acid levels in CSF samples may provide a means of determining prognosis at an early stage and extending the knowledge about the pathophysiology of SAH.

## Materials and Methods

### Ethics and Consent

This is a prospective observational study conducted in a single medical centre in accordance with the Declaration of Helsinki. The local bioethics committee approved the study protocol, consenting protocol, and consent forms. Patients were assessed by two specialists (neurosurgeon and anaesthesiologist) as to their ability to give informed consent. Depending on this assessment, either the patient or next of kin gave consent for entry to the study and use a blinded medical data for analysis and further publication.

### Population, Inclusion and Exclusion Criteria

132 patients with SAH [confirmed by non-contrast computed tomography (CT)] were referred to our department during study recruitment period (from May 2015 to October 2016). Inclusion criteria were as follows: (1) aneurysmal SAH treated endovascularly <24 h post rupture, (2) external ventricular drainage (EVD) placed <48 h post rupture. The aim was early prevention of rebleeding while managing acute hydrocephalus (HCP), but avoiding the trauma associated with open surgery. Exclusion criteria for the study were as follows: (1) history of CNS disease (meningitis, stroke), (2) active CNS infection, (3) active systemic disease (diabetes mellitus, rheumatoid arthritis, malignancy, cirrhosis, renal failure), (4) age below 18, and (5) pregnancy. Conditions with potential impact on CSF homeostasis as well as subpopulations with distinct SAH features were not enrolled. Control CSF was obtained during spinal anaesthesia from age- and sex-matched patients with a negative history of CNS diseases.

### Management, Definitions, and End Points

On admission, the clinical status was assessed using GCS and specific SAH grading scales [Hunt and Hess (HH), World Federation of Neurosurgical Societies (WFNS)]. An initial head CT scan was used to confirm SAH and assess the presence of HCP. EVD was placed secondary to endovascular treatment in patients with GCS score below 15 and: (1) relative bicaudate index >1, (2) focal dilation of ventricular system due to obstruction, or (3) thick intraventricular blood clot. A second CT scan was performed within 24 h of aneurysmal occlusion and EVD placement to assess any procedure related brain injury. Patients received a continuous infusion of nimodipine for at least 10 days, hypotension was avoided using vasopressors, and euvolemia was maintained. Induced hypertension (20–30% above baseline levels) was used to treat patients diagnosed with delayed cerebral ischaemia (DCI), based on the appearance of a new focal deficit, or a drop of at least two points on the GCS lasting at least 2 h after the exclusion of systematic causes EVD infection screening involved CSF cell count at least twice per patient, and CSF culture at least once on day 10 post-SAH. The primary end point was the treatment outcome assessed at 3 months using the Glasgow Outcome Scale (GOS) (22). Patients were divided into two groups according to GOS. Good outcome consisted of those with no disability, moderate disability, and severe disability (GOS grades 5, 4, and 3); poor outcome were those with persistent vegetative state or death (GOS grades 2 and 1). DCI related infarction was defined as a new cerebral infarction identified on a head CT scan within 6 weeks of rupture and not present on the immediate posttreatment scan [as proposed by Vergouwen et al. (23)].

### Plasma Assays and CSF Sample Collection

Haemoglobin, white blood cell (WBC) count, C-reactive protein (CRP) level, and fibrinogen level were assessed daily. Automatic analysers XT 2000i (Sysmex, Japan), Cobas 6000 (Roche Diagnostic, USA), and ACL TOP 500 (Instrumentation Laboratory, Italy) were used for measurements. CSF samples were collected from the EVD at three time points, on post-SAH days 0–3, 5, and 10. Each CSF sample was centrifugated and stored at −80°C until assayed.

### Determination of Free Amino Acid Profiles

The applied methodology was based on an aTRAQ™ kit (Sciex, Framingham, MA, USA). The detailed description of a sample preparation procedure as well as liquid chromatography–mass spectrometry/mass spectrometry (LC–MS/MS) parameters have been described in our previous report (24). The sample preparation comprised the following steps: protein precipitation by 10% sulphosalicyclic acid, dilution with borate buffer, amino acids labelling with aTRAQ™ reagent, and addition of internal standard mixture. Labelling efficiency was confirmed using norleucine (contained in sulphosalicylic acid solution) and norvaline (contained in borate buffer). LC–MS/MS assays were carried out on a high-performance liquid chromatography instrument 1260 Infinity (Agilent Technologies, Santa Clara, CA, USA) coupled with 4000 QTRAP triple quadrupole mass spectrometer (Sciex, Framingham, MA, USA). Chromatographic separation of amino acids was performed using a Sciex C18 column (4.6 mm × 150 mm, 5 µm) and a flow rate of 800 µL/min in a gradient elution mode. Solvent A was water and solvent B was methanol, both with 0.1% formic acid and 0.01% heptafluorobutyric acid. The injection volume was 2 µL. The mass spectrometer was equipped with an electrospray ionisation source and operated in positive ionisation mode. For detection and quantification of amino acids, a highly selective schedule multiple reaction monitoring mode was used. Data acquisition and processing were performed with Analyst 1.5 software (Sciex, Framingham, MA, USA). The applied methodology is suitable for simultaneous determination of a wide range of 33 free amino acids, both proteinogenic and non-proteionogenic, with high sensitivity and specificity in time below 20 min (24–26). Due to simplification used in this article, *o*-phosphoethanolamine and ethylamine are counted to amino acids, yet in fact they are amino acids derivatives.

### Statistical Analysis

Statistical analysis was performed using STATISTICA 10 (Stat Soft Inc., Tulsa, OK, USA). Values for normally distributed numerical data have been expressed as mean and SDs; for ordinal or non-normally distributed numerical data as median and interquartile range, and for categorical data as counts and percentages. The normality of data distribution was assessed using the Shapiro–Wilk test. Amino acids levels are presented on figures as median and interquartile range since in nearly all cases they do not show normal distribution. Comparisons were made by using (1) Mann–Whitney test, (2) Student’s *t*-test, (3) Friedman test with Conover–Iman *post hoc*, and (4) repeated measures ANOVA test with Fisher *post hoc*. The correlations were assessed by Spearman’s test, and correlation coefficient (cc) >0.6 (cc < −0.6) was considered significant. A value of *p* < 0.05 was considered statistically significant when comparing.

## Results

The concentrations of the 33 compounds were established for 49 samples derived from 23 SAH patients (Table 1), and 25 samples collected from 25 control patients. Assays were available from 22 samples at days 0–3 post-SAH, 15 from days 5, and 12 from day 10. Rupture of an aneurysm led to a significant elevation of 27 of the 33 compounds in the CSF at days 0–3 post-SAH (Table 2). On days 0–3 post-SAH, the six exceptions were ethylamine (*p* = 0.798), gamma-aminobutyric acid (*p* = 0.699), 3-amino-isobutyric acid (*p* = 0.668), hydroxyproline (*p* = 0.164), threonine (*p* = 0.079), and arginine (*p* = 0.062). On day 5 post-SAH, three compounds showed no significant difference—aspartic acid (*p* = 0.175), 3-amino-isobutyric acid (*p* = 0.563), and hydroxyproline (*p* = 0.658). On day 10 post-SAH, there were four—aspartic acid (*p* = 0.054), 3-amino-isobutyric acid (*p* = 0.806), hydroxyproline (*p* = 0.289), and ethylamine (*p* = 0.292). Thus, hydroxyproline and 3-amino-isobutyric acid are the only 2 of the 33 substances assayed which appear to show no increase following SAH.

**Table 1:** Baseline characteristics of study patients.

Male	12 (52%)		
Age (years)	57.26 ± 14.78		
**Aneurysm location**			
Anterior communicating artery	8 (35%)		
Middle cerebral artery	6 (26%)		
Anterior cerebral artery	3 (13%)		
Basilar artery	3 (13%)		
Internal carotid artery	1 (4%)		
Posterior cerebral artery	1 (4%)		
Posterior inferior cerebellar artery	1 (4%)		
Aneurysmal size (mm)	4.74 ± 1.82		
Cerebral infarction due to DCI on CT	15 (65%)		
Intracerebral haemorrhage on CT	15 (65%)		
Intraventricular blood on CT	21 (91%)		
Fisher CT score	4 (4–4)		
Modified Fisher CT score	4 (3–4)		
WFNS score on admission	4 (3–5)		
HH score on admission	4 (3–5)		
GCS on admission	5 (4–12)		

	**On admission**	**Post-SAH day 5**	**Post-SAH day 10**

WBC count (10^6^/mm^3^)	13.69 ± 5.4	11.86 ± 5.7	12.51 ± 4.3
CRP level (mg/L)	119.50 ± 79.5	89.42 ± 54.4	78.70 ± 119
Fibrinogen (mg/dL)	446.70 ± 151	608.20 ± 205	592.90 ± 249
Hgb (g/dL)	16.40 ± 17.41	11.31 ± 1.9	10.93 ± 1.3
**Treatment outcome (according to GOS at 3 months)**			
Good recovery (score of 5)	6 (26%)		
Moderate disability (score of 4)	2 (9%)		
Severe disability (score of 3)	2 (9%)		
Persistent vegetative state (score of 2)	5 (22%)		
Death (score of 1)	8 (35%)		

*Data presented as mean ± SD; median (interquartile range) or count (percentage)*.

*CRP, C-reactive protein; CT, computed tomography; DCI, delayed cerebral ischaemia; GCS, Glasgow Coma Scale; GOS, Glasgow Outcome Scale; HH, Hunt and Hess scale; Hgb, plasma haemoglobin; WBC, white blood cell; WFNS, World Federation of Neurosurgical Societies scale*.

**Table 2:** Differences in cerebrospinal fluid amino acid level at days 0–3, 5, and 10 post-SAH in healthy individuals (control group) and subarachnoid haemorrhage (SAH) patients (study group).

Amino acid	Control group	Study group, day 0–3 post-SAH	Control group vs study group, days 0–3 post-SAH. *p* value	Study group, day 5 post-SAH	Control group vs study group, day 5 post-SAH. *p* value	Study group, day 10 post-SAH	Control group vs study group, day 10 post-SAH. *p* value
O-phosphoethanolamine (μM)	3.00	5.95	**<0.01**	5.15	**<0.001**	6.60	**<0.001**
Ethylamine (μM)	9.80	9.40	0.798	9.65	**<0.001**	11.10	0.292
Taurine (μM)	7.00	16.00	**<0.001**	14.30	**<0.001**	10.75	**<0.01**
Asparagine (μM)	5.70	10.35	**0.01**	19.50	**<0.001**	21.75	**<0.001**
Serine (μM)	24.20	55.70	**<0.001**	75.40	**<0.001**	83.75	**<0.001**
Glycine (μM)	8.90	38.75	**<0.001**	34.85	**<0.001**	35.20	**<0.001**
Hydroxyproline (μM)	0.60	1.00	0.164	1.35	0.658	1.35	0.289
Glutamine (μM)	407.60	520.65	**<0.01**	791.10	**<0.001**	796.30	**<0.001**
Aspartic acid (μM)	0.50	1.55	**<0.001**	0.80	0.175	0.95	0.054
Citruline (μM)	1.60	2.75	**0.011**	2.55	**<0.01**	3.30	**<0.01**
Threonine (μM)	24.80	29.05	0.079	56.10	**<0.001**	63.40	**<0.001**
Beta-alanine (μM)	20.30	22.00	**0.040**	29.00	**<0.01**	27.30	**<0.01**
Alanine (μM)	35.00	88.05	**<0.001**	114.55	**<0.001**	122.55	**<0.001**
Glutamic acid (μM)	1.20	5.40	**<0.001**	3.00	**< 0.001**	2.35	**<0.01**
Histidine (μM)	11.40	28.20	**<0.001**	43.35	**<0.001**	40.20	**<0.001**
3-Methylhistidine (μM)	0.50	1.00	**<0.01**	1.40	**<0.001**	1.05	**<0.01**
2-Aminoadipic acid (μM)	0.00	1.65	**<0.001**	2.10	**<0.001**	1.45	**<0.001**
Gamma-aminobutyric acid (μM)	0.40	0.50	0.699	0.30	**0.012**	0.20	0.051
3-Aminoisobutyric acid (μM)	0.50	0.30	0.668	0.30	0.563	0.35	0.806
2-Aminobutyric acid (μM)	3.20	6.20	**<0.01**	6.60	**<0.001**	7.30	**<0.001**
Arginine (μM)	15.70	19.25	0.062	26.60	**<0.001**	24.35	**<0.01**
Proline (μM)	0.80	11.80	**<0.001**	13.95	**<0.001**	16.15	**<0.001**
Ornithine (μM)	4.30	19.80	**<0.001**	17.95	**<0.001**	17.75	**<0.001**
Cystathionine (μM)	0.30	2.05	**<0.001**	1.05	**<0.001**	1.40	**<0.001**
Cysteine (μM)	0.40	2.05	**<0.001**	1.75	**<0.001**	1.65	**<0.001**
Lysine (μM)	22.50	53.80	**<0.001**	73.05	**<0.001**	72.40	**<0.001**
Methionine (μM)	3.10	6.20	**<0.01**	11.70	**<0.001**	13.65	**<0.001**
Valine (μM)	16.20	38.65	**<0.01**	66.20	**<0.001**	76.30	**<0.001**
Tyrosine (μM)	7.80	26.70	**<0.001**	36.20	**<0.001**	37.30	**<0.001**
Isoleucine (μM)	4.40	9.65	**0.001**	13.05	**<0.001**	15.05	**<0.001**
Leucine (μM)	11.40	29.20	**<0.001**	43.50	**<0.001**	56.80	**<0.001**
Phenylalanine (μM)	9.40	26.45	**<0.001**	43.25	**<0.001**	44.45	**<0.001**
Tryptophan (μM)	1.90	9.70	**<0.001**	13.60	**<0.001**	12.85	**<0.001**

*Median levels of the amino acids are presented in the table. *p* values were calculated either by Mann–Whitney or Student’s *t*-test. Amino acids increasing significantly during SAH (on days 0–3, 5, or 10) are shown in bold*.

**Figure 1:**
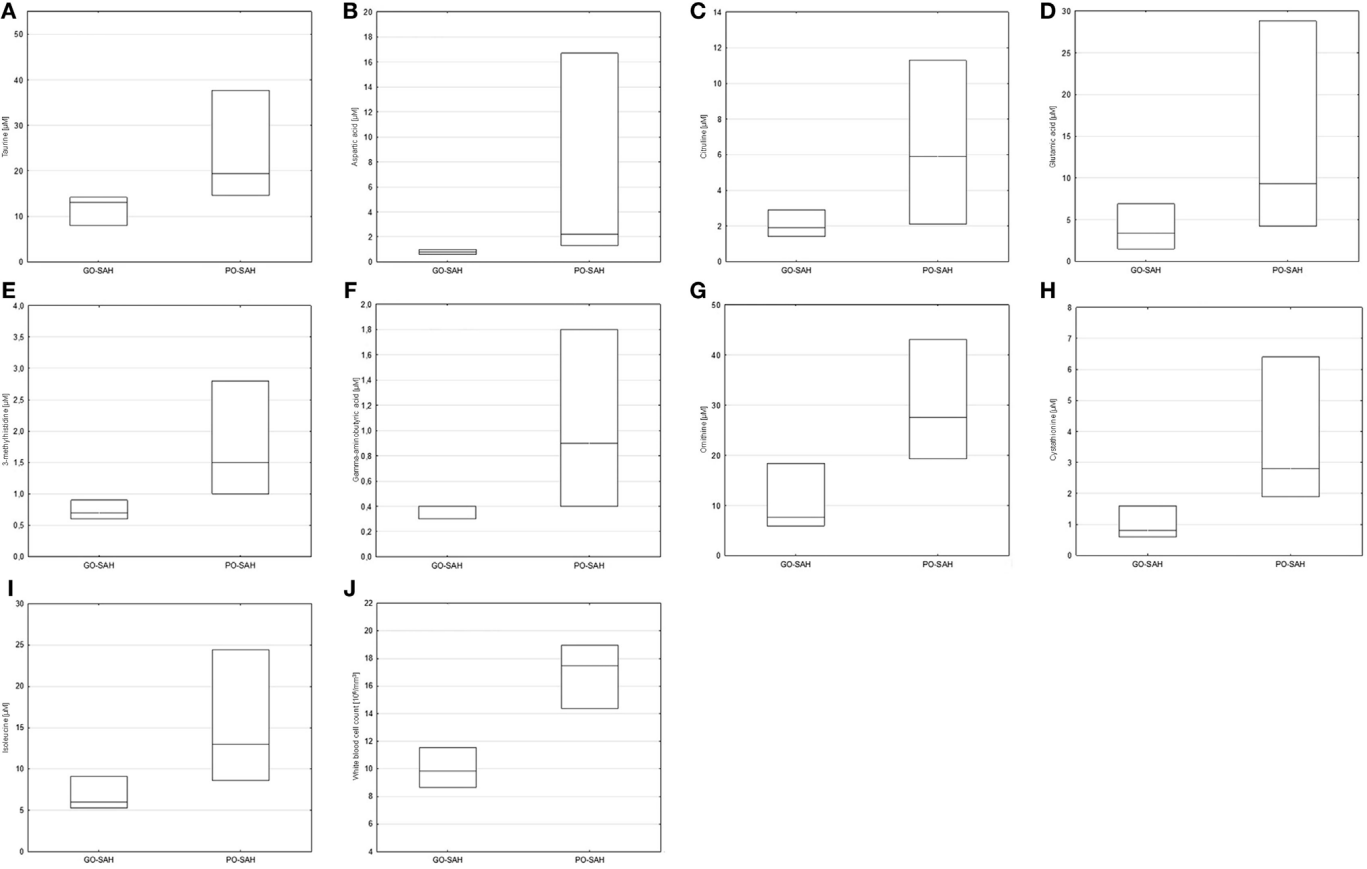
Significant differences in monitored parameters on days 0–3 post-SAH between patients with good (GO-SAH) and poor (PO-SAH) treatment outcome. Mann–Whitney test revealed significantly higher levels of **(A)** taurine (*p* = 0.038), **(B)** aspartic acid (*p* = 0.038), **(C)** citruline (*p* = 0.035), **(D)** glutamic acid (*p* = 0.038), **(E)** 3-methylhistidine (*p* < 0.01), **(F)** gamma-aminobutyric acid (*p* = 0.043), **(G)** ornithine (*p* = 0.033), **(H)** cystathionine (*p* < 0.01), and **(I)** isoleucine (*p* = 0.045) in patients with poor outcome. Student’s *t*-test revealed significantly higher level of white blood cell count (*p* < 0.01) in patients with poor outcome **(J)**. In all cases, median levels and the 25th and 75th percentiles are presented.

**Figure 2:**
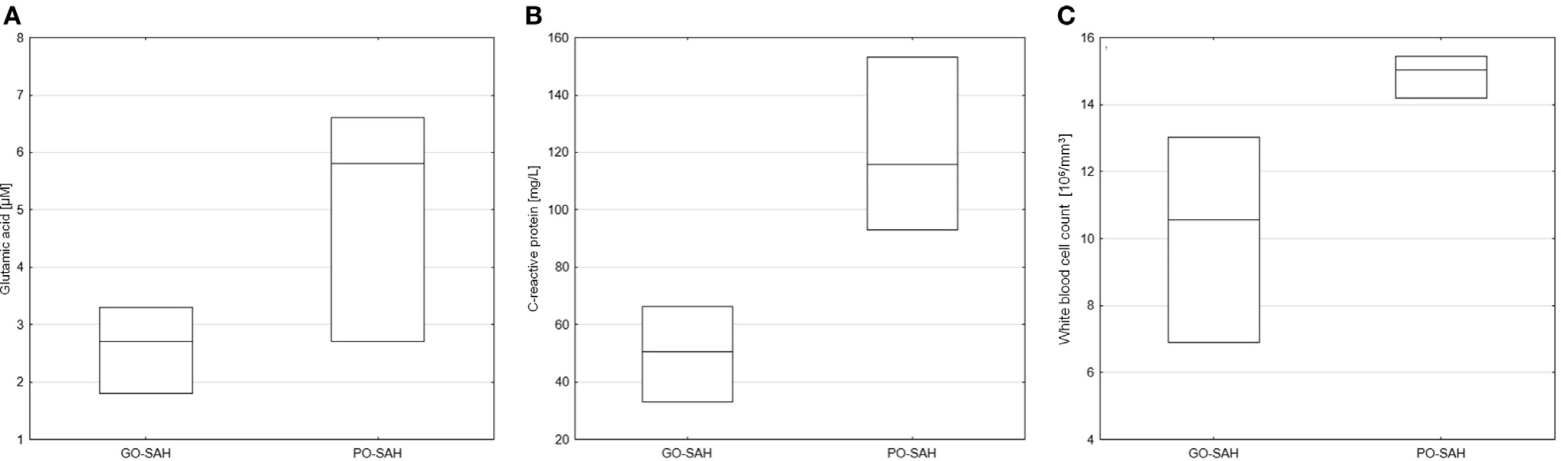
Significant differences in monitored parameters on day 5 post-SAH between patients with good (GO-SAH) and poor (PO-SAH) treatment outcome. **(A)** Student’s *t*-test revealed significantly higher levels of glutamic acid (*p* = 0.041) in patients with poor outcome. Mann–Whitney test revealed significantly higher C-reactive protein level (*p* 0.020) **(B)** and white blood cell count (*p* = 0.044) **(C)** in patients with poor outcome. In all cases, median levels and the 25th and 75th percentiles are presented.

**Figure 3:**
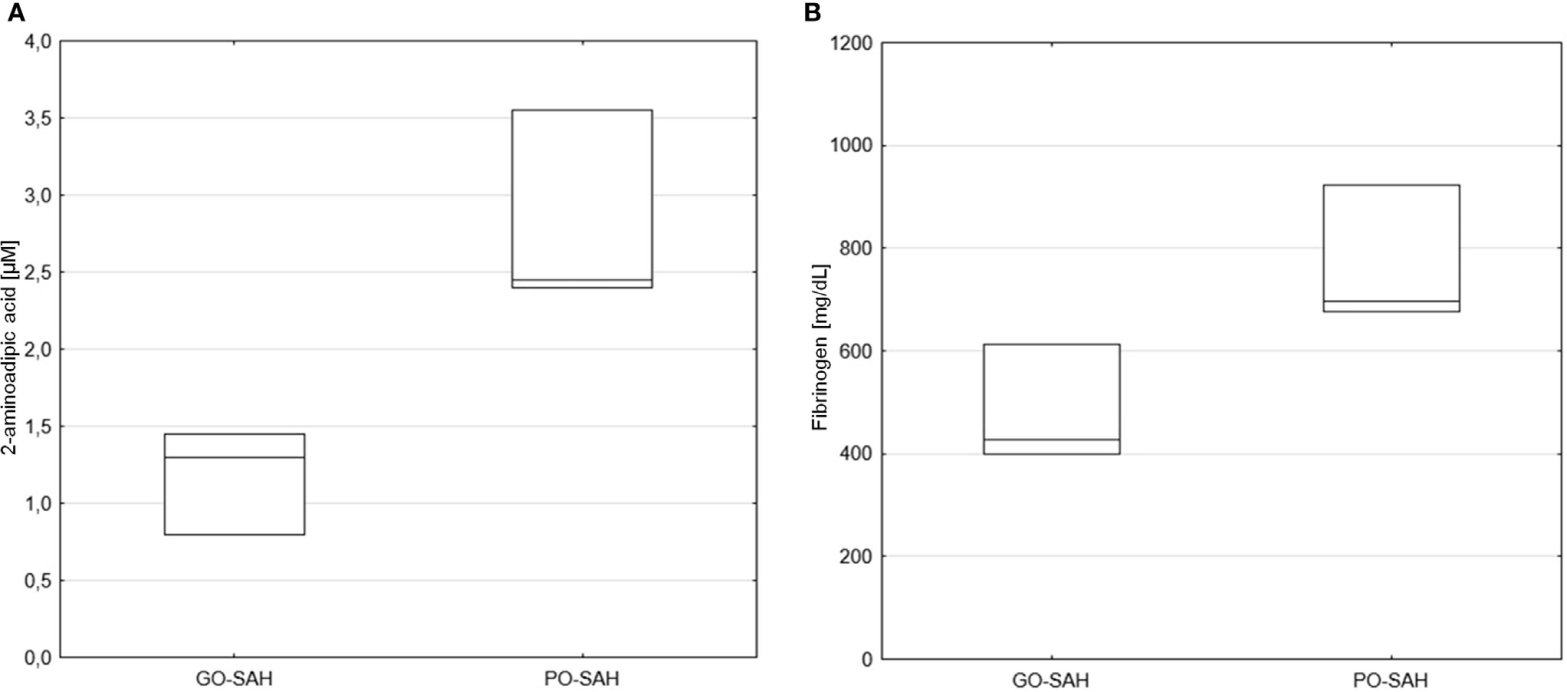
Significant differences in monitored parameters on day 10 post-SAH between patients with good (GO-SAH) and poor (PO-SAH) treatment outcome. Mann–Whitney test revealed significantly higher 2-aminoadipic acid (*p* = 0.033) **(A)** and fibrinogen (*p* = 0.014) **(B)** levels in patients with poor outcome. Median levels and the 25th and 75th percentiles are presented.

Patients were now divided into two groups as good and poor outcomes as defined above (Table 3). On days 0–3 post-SAH, concentrations of nine amino acids were significantly higher in patients with poor outcome than those with good outcome: taurine (*p* = 0.038), aspartic acid (*p* = 0.038), citrulline (*p* = 0.035), glutamic acid (*p* = 0.038), gamma-amino-butyric acid (*p* = 0.043), 3-methyl-histidine (*p* = 0.01), ornithine (*p* = 0.033), cystathionine (*p* = 0.01), and isoleucine (*p* = 0.045). WBC level (*p* = 0.01) also differentiated these two groups (Figure 1). On day 5 post-SAH, glutamic acid (*p* = 0.041) was the only amino acid showing significantly higher levels in the poor outcome group. At this stage, CRP (*p* = 0.020) and WBC (*p* = 0.044) were also significantly higher in the poor outcome group (Figure 2). On day 10 post-SAH, 2-amino-adipic acid (*p* = 0.033) and fibrinogen (*p* = 0.014) were the only parameters showing significantly higher levels in the poor outcome group (Figure 3).

**Table 3:** Differences in monitored parameters at days 0–3, 5, and 10 post-SAH in patients with good (GO-SAH) and poor (PO-SAH) treatment outcome.

Monitored parameter	Days 0–3 post-SAH			Day 5 post-SAH			Day 10 post-SAH		
	GO-SAH	PO-SAH	GO-SAH vs PO-SAH. *p* value	GO-SAH	PO-SAH	GO-SAH vs PO-SAH. *p* value	GO-SAH	PO-SAH	GO-SAH vs PO-SAH. *p* value
C-reactive protein (mg/L)	125.00	130.00	0.715	50.70	115.80	**0.020**	22.40	109.70	0.139
White blood cell count (10^6^/mm^3^)	9.86	17.47	**<0.001**	10.57	15.03	**0.044**	9.66	16.84	0.445
Haemoglobin (g/dL)	12.10	13.10	0.212	11.30	10.90	0.262	10.60	11.50	0.672
Temperature (°C)	37.00	37.00	0.736	37.40	37.20	0.273	36.90	35.70	0.512
Fibrinogen (mg/dL)	485.00	550.00	0.879	509.00	676.00	0.257	428.00	698.00	**0.014**
O-phosphoethanolamine (μM)	4.10	6.80	0.161	4.90	7.70	0.161	5.95	8.50	0.019
Taurine (μM)	13.10	19.40	**0.038**	14.60	13.40	0.514	12.20	7.35	0.138
Asparagine (μM)	9.00	11.50	0.256	30.20	12.50	0.397	22.90	20.45	0.423
Serine (μM)	45.10	76.50	0.161	77.00	61.40	0.947	79.50	94.75	0.503
Glycine (μM)	29.60	41.00	0.182	30.00	43.00	0.204	35.20	35.65	0.671
Hydroxyproline (μM)	0.70	1.30	0.066	1.70	0.80	0.518	1.35	1.10	0.793
Ethylaminev (μM)	5.80	15.80	0.102	7.90	12.10	0.134	8.85	14.50	0.35
Glutamine (μM)	489.90	602.50	0.182	837.30	505.10	0.585	852.50	715.60	0.483
Aspartic acid (μM)	0.80	2.20	**0.038**	0.40	1.00	0.071	0.95	1.30	0.551
Citruline (μM)	1.90	5.90	**0.035**	2.50	3.10	0.396	3.30	3.30	1
Threonine (μM)	25.90	50.00	0.35	86.30	34.20	0.327	63.40	57.95	0.298
Beta-alanine (μM)	28.30	22.00	0.07	30.00	28.60	0.497	26.85	27.85	0.893
Alanine (μM)	70.70	138.10	0.109	115.50	113.60	0.447	120.30	141.70	0.954
Glutamic acid (μM)	3.40	9.30	**0.038**	2.70	5.80	**0.024**	2.00	17.10	0.269
Histidine (μM)	18.90	38.10	0.256	63.60	23.70	0.711	43.55	37.45	0.753
3-Methylhistidine (μM)	0.70	1.50	**<0.01**	1.80	1.20	0.958	1.35	1.05	0.733
2-Aminoadipic acid (μM)	1.30	1.70	0.076	1.50	4.10	0.071	1.30	2.45	**0.033**
Gamma-aminobutyric acid (μM)	0.30	0.90	**0.043**	0.30	0.30	0.932	0.35	0.15	0.266
3-Aminoisobutyric acid (μM)	0.20	0.40	0.066	0.30	0.30	0.958	0.35	0.45	0.93
2-Aminobutyric acid	4.60	8.20	0.088	11.40	5.50	0.542	10.75	6.50	0.124
Arginine (μM)	18.50	22.30	0.182	30.90	26.00	0.525	24.35	20.45	0.21
Proline (μM)	4.50	14.80	0.204	18.70	10.90	0.672	16.15	18.40	0.759
Ornithine (μM)	7.60	27.50	**0.033**	17.30	28.30	0.09	17.35	33.05	0.552
Cystathionine (μM)	0.80	2.80	**<0.01**	1.00	1.80	0.089	1.30	1.50	0.199
Cysteine (μM)	1.60	2.30	0.141	1.50	2.20	0.243	1.95	1.40	0.329
Lysine (μM)	43.00	74.50	0.095	87.10	55.90	0.341	72.40	72.60	0.612
Methionine (μM)	4.60	12.30	0.204	16.20	7.70	0.491	13.65	13.70	0.687
Valine (μM)	31.80	62.40	0.109	102.50	33.60	0.397	87.75	72.20	0.377
Tyrosine (μM)	23.00	32.50	0.083	36.60	35.80	0.876	38.60	37.30	0.676
Isoleucine (μM)	6.00	13.00	**0.045**	17.60	11.80	0.597	15.05	13.70	0.45
Leucine (μM)	22.90	39.70	0.109	80.30	29.80	0.397	60.95	50.55	0.532
Phenylalanine (μM)	21.20	38.80	0.062	51.30	32.40	0.665	45.40	44.45	0.913
Tryptophan (μM)	8.00	12.20	0.066	13.60	13.60	0.606	12.60	13.55	0.804

*Median levels of the amino acids are presented in the table. p values were calculated either by Mann–Whitney or Student’s t-test. Significant differences are shown in bold*.

In the course of the study, it was possible to undertake assays at all three time points in 10 of the subjects; 7 of these were in the good outcome group. In this group, the Friedman test with Conover–Iman *post hoc*, or ANOVA test with Fisher *post hoc*, revealed significant changes in the concentrations of 18 of the compounds tested (Table 4). The number of poor outcome patients was too small to carry out such testing.

**Table 4:** Amino acid level changes in time in good outcome SAH patients with (GO-SAH).

Monitored parameter	Days 0–3 post-SAH	Day 5 post-SAH	Day 10 post-SAH	Omnibus test *p* value	Days 0–3 vs Day 5 *p* value *post hoc*	Days 0–3 vs Day 10 *p* value *post hoc*	Day 5 vs Day 10 *p* value *post hoc*
C-reactive protein (mg/L)	125.00	50.70	27.00	**0.049**	0.345	**0.012**	0.073
White blood cell count (10^6^/mm^3^)	9.86	10.57	9.34	0.631			
Haemoglobin (g/dL)	12.10	10.60	10.50	0.066			
Temperature (°C)	37.00	37.30	36.90	0.382			
Fibrinogen (mg/dL)	485.00	442.00	423.00	0.327			
O-phosphoethanolamine (μM)	6.20	5.00	5.80	0.325			
Taurine (μM)	13.90	14.60	10.80	0.867			
Asparagine (μM)	8.40	18.30	21.20	0.180			
Serine (μM)	45.10	73.80	76.10	**0.031**	**0.039**	**0.013**	0.566
Glycine (μM)	29.60	32.60	42.50	0.565			
Hydroxyproline (μM)	0.70	1.10	1.40	0.094			
Ethylaminev (μM)	5.70	10.10	9.80	**0.039**	0.078	**<0.01**	0.187
Glutamine (μM)	454.30	818.70	828.30	**0.001**	**<0.01**	**<0.001**	0.565
Aspartic acid (μM)	0.70	0.40	1.20	0.215			
Citruline (μM)	1.90	2.40	2.80	0.069			
Threonine (μM)	22.90	52.50	61.20	**0.012**	0.128	**<0.01**	**<0.01**
Beta-alanine (μM)	28.30	26.80	26.70	0.553			
Alanine (μM)	70.70	106.20	120.30	0.124			
Glutamic acid (μM)	4.50	2.70	2.10	0.898			
Histidine (μM)	16.80	38.10	37.40	0.156			
3-Methylhistidine (μM)	0.60	1.50	0.90	0.369			
2-Aminoadipic acid (μM)	1.30	1.50	1.30	0.129			
Gamma-aminobutyric acid (μM)	0.30	0.20	0.50	0.633			
3-Aminoisobutyric acid (μM)	0.20	0.30	0.30	0.215			
2-Aminobutyric acid (μM)	4.60	11.40	11.40	**0.010**	**0.019**	**<0.01**	0.427
Arginine (μM)	18.50	26.50	24.10	**0.050**	0.073	**0.012**	0.345
Proline (μM)	3.40	11.90	15.20	0.062			
Ornithine (μM)	7.60	17.30	14.30	0.368			
Cystathionine (μM)	1.00	1.00	1.20	0.648			
Cysteine (μM)	1.60	1.70	2.10	0.368			
Lysine (μM)	43.00	83.20	70.80	**0.017**	**0.017**	**<0.01**	0.735
Methionine (μM)	4.60	10.00	11.90	**0.008**	**0.029**	**<0.01**	0.200
Valine (μM)	28.20	61.60	78.10	**0.015**	**0.017**	**<0.01**	0.683
Tyrosine (μM)	23.00	35.10	28.50	**0.020**	**0.023**	**<0.01**	0.647
Isoleucine (μM)	5.90	11.10	13.60	**0.024**	0.081	**<0.01**	0.219
Leucine (μM)	22.60	39.70	60.50	**0.024**	**0.022**	**0.013**	0.801
Phenylalanine (μM)	20.90	35.20	37.50	**0.038**	**0.040**	**0.018**	0.700
Tryptophan (μM)	8.00	11.80	11.10	0.072			

*Median levels of the amino acids are presented in the table. Omnibus statistical test (Friedman or repeated measures ANOVA) revealed significant changes in concentration of 13 amino acids (shown in bold). Next, *post hoc* analysis (Conover–Iman for Friedman test and Fisher for repeated measures ANOVA) was run for those 13 amino acids to specify between what time points (days 0–3, 5, or 10 post-SAH) the differences in concentration were significant (shown in bold)*.

**Figure 4:**
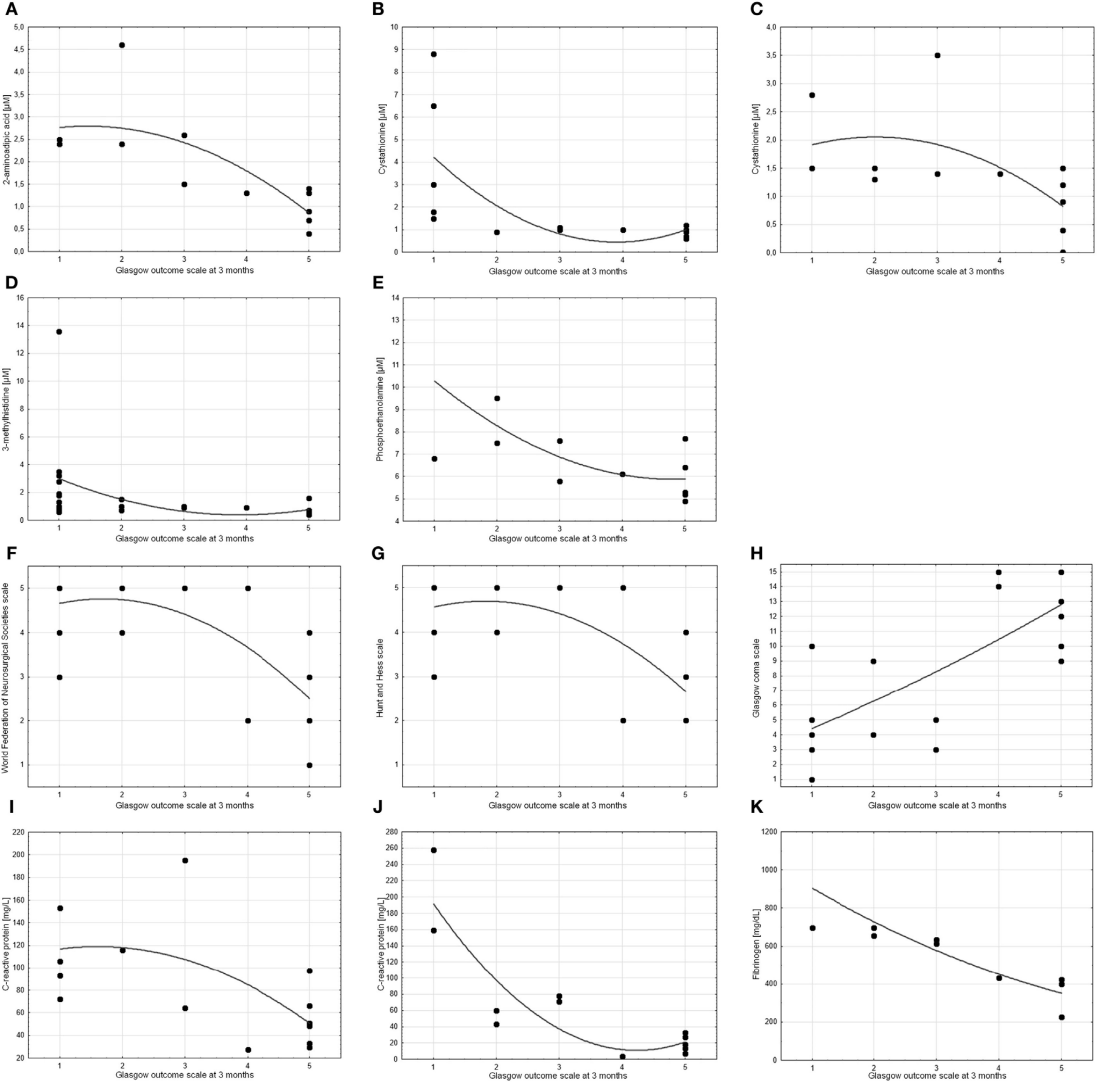
Scatter charts showing the correlation between treatment outcome (measured by Glasgow Outcome Scale at 3 months) and other parameters. Spearman’s test revealed significant correlations (cc > 0.6 or cc < −0.6) for **(A)** 2-aminoadipic acid on day 10 post-SAH (cc = −0.81), **(B,C)** cystathionine on day 5 post-SAH (cc = −0.72) and day 10 post-SAH (cc = −0.67), **(D)** 3-methylhistidine on days 0–3 post-SAH (cc = −0.64), **(E)** *o*-phosphoethanolamine on day 10 post-SAH (cc = −0.62), **(F)** World Federation of Neurosurgical Societies scale (cc = −0.64), **(G)** Hunt and Hess scale (cc = −0.61), **(H)** Glasgow coma scale (cc = 0.72), **(I,J)** C-reactive protein on day 5 post-SAH (cc = −0.64) and day 10 post-SAH (cc = −0.79), and **(K)** fibrinogen on day 10 post-SAH (cc = −0.97). *p* values are <0.05 in all cases.

The levels of four of the compounds tested showed a significant correlation with GOS at 3 months. These were 2-amino-adipic acid on day 10 post-SAH (cc = −0.81), cystathionine on day 5 post-SAH (cc = −0.72) and day 10 post-SAH (cc = −0.67), 3-methylhistidine on days 0–3 post-SAH (cc = −0.64), and *o*-phosphoethanolamine on day 10 post-SAH (cc = −0.62). As might be expected, there was a correlation between all three admission assessments (WFNS, HH, and GCS) and outcome. There were also correlations between outcome and CRP on days 5 and 10 post-SAH (cc = −0.64 and cc = −0.79, respectively) as well as fibrinogen level on day 10 post-SAH (cc = −0.97) (Figure 4).

## Discussion

In this study, we have analysed the profiles of amino acids and related compounds in the CSF of patients following SAH. The most significant findings are as follows:

(1) Increase in 31 of 33 compounds in the days following SAH, with 26 increasing in the first 3 days after rupture
(2) Significant increase in 18 of these compounds between days 0–3 and days 5 and 10
(3) Higher levels of excitatory amino acids (EAAs) (glutamic acid, aspartic acid, and 2-amino-adipic acid) appear to predict a poor outcome.

As far as we are aware, it is 31 years since an article investigating amino acids in SAH has been published. In view of the paucity of publications, knowledge of this subject remains rudimentary. With the introduction of microdialysis, interest in this subject has been renewed since it allows *in vivo* sampling of brain interstitial fluid (27). This method allows continuous monitoring of brain metabolism, but this is limited to the tissue around the probe (28). On the other hand, the commonly available CSF analysis gives a more general picture of conditions in the brain. Physiologically, the exchange between brain interstitial fluid and CSF is bidirectional (29). In mice, levels of amino acids in brain interstitial fluid were found to be approximately 5–10 times lower than in the CSF (30). Although the latest studies of amino acids in SAH have focussed on microdialysis, widespread use of this monitoring technique is limited by its high cost. By contrast, EVD is a relatively inexpensive procedure commonly performed in patients following SAH. In the clinical setting, CSF seems to be a more convenient source for examining biomarkers.

In our patients, rupture of an aneurysm led to an increase in 31 of 33 amino acids and related compounds we assayed. In the study by von Holst et al., there was no increase in taurine levels following SAH. Because the concentration of taurine in whole blood is four to eight times greater than in blood plasma alone, von Holst et al. concluded that red blood cell (RBC) lysis did not contribute to the amino acid concentrations (31). We do not agree with this statement as in our series, the CSF taurine levels increased by a factor of 2. RBC lysis begins within 2–4 h of SAH and continues at least until the clot has cleared (32, 33). In our opinion, this process contributes to the amino acid levels at every stage following SAH. Microdialysis studies indicate that an early increase of taurine in the brain interstitial fluid is a reliable marker of poor outcome (34, 35). In the both articles, brain cells activated in the course of SAH were indicated as a potential source of taurine in the interstitial fluid. In our series, significantly higher taurine levels were observed on days 0–3 post-SAH in patients with a poor outcome. This observation suggests that taurine may have some value as a clinical marker and encourage further studies. In experimental settings, both detrimental and beneficial roles for taurine have been described. Kofler et al. have extensively discussed this matter in the context of SAH (34). Our study suggests predominantly harmful effects from taurine.

Among 33 assayed compounds, only 2 did not increase at any stage; these were hydroxyproline and 3-aminoisobutyric acid, neither of which is encoded in the eukaryotic genetic code. Hydroxyproline is produced by hydroxylation of proline and incorporated into collagen protein (36). In patients with blood–CSF barrier dysfunction, hydroxyproline has a smaller biological variation in CSF when compared with other amino acids (37). Hydroxyproline is increased in the blood plasma in Alzheimer’s disease, but shows no increase in Parkinson’s disease (38, 39). High protein bound of hydroksyproline in human erythrocytes could be an explanation of its elevation absence in CSF following SAH (40). Even less data are available for 3-aminoisobutyric acid, although it is known that it is a product of thymine catabolism and plays a role in fatty acid metabolism (41). The alterations of 3-aminoisobutyric acid could be a potential marker for the monitoring of the blood–brain barrier condition in the future studies (42).

Immediately after aneurysmal rupture, significantly higher levels of nine of the compounds investigated identified the patients with a poorer prognosis. Furthermore, none of the substances showed a significant decrease in CSF following SAH. We suspect that the initial increase is due to extravasated blood, and its extent related to the amount of blood. The main source is likely to be plasma, since the majority of amino acids (except glutamine and glutamic acid) have a CSF:plasma ratio of 0.1–0.2 (43). This is consistent with the fact that patients with a greater volume of subarachnoid blood have a poorer outcome (44).

In 18 of the compounds we investigated, there was a significant increase from days 0–3 post-SAH to days 5 and 10. There are several possible mechanisms for this delayed rise: (1) increased amino acid turnover as a response to injury. Zetterling et al. proposed this mechanism as an explanation for an increase in the concentrations of eight non-transmitter amino acids in brain interstitial fluid (45). (2) Cytokine-stimulated amino acid release. High-mobility group box 1 protein (HMGB1), which is a proinflammatory cytokine [found in CSF of SAH patients (46)], which induces the release of the glutamate analogue from gliosomes (glial resealed subcellular particles) in a concentration-dependent manner (47). Conversely, HMGB1 was found to accumulate in glutamate treated primary cortical culture media, and supernatants collected from these cultures were found to trigger microglial activation (48). (3) Disruption of CSF homeostasis affecting transport with both the bloodstream and the interstitial fluid. (4) Lysis of RBCs (mentioned above) subsequent to their continuous release from the clot during its clearance (33).

Glutamic acid, as an important EAA, is the most extensively studied amino acid in SAH. Levels of glutamic acid in the interstitial fluid increase within minutes of SAH, peak at 40 min, and remain elevated for days (49). The increase is most pronounced in patients with acute ischaemic neurological deficit (50). Increase in interstitial glutamic acid was identified as one of the earliest markers of impending ischaemia, typically increasing before the onset of clinical symptoms (27, 50). Our results are in line with these observations. Glutamic acid, aspartic acid, and 2-aminoadipic acid levels increase on days 0–3 post-SAH, and are all significantly higher at some point in the poor outcomes group of patients. In fact, in this group, outcome was most accurately predicted by 2-aminoadipic acid levels in CSF on day 10 post-SAH. 2-Aminoadipic acid is a structural homologue of glutamic acid and a natural product of lysine metabolism in mammalian cells (51). Huck et al. described gliotoxic properties of this amino acid (52), while Kato et al. observed enhanced susceptibility of glial cell to oxidative stress after 2-aminoadipic acid administration (53).

In this prospective observational study, some limitations need to be considered. First, specific enrolment criteria (acute HCP and EVD insertion) interfere with typical SAH pathophysiology. They will aggravate the SAH-associated brain injury and may well alter amino acid concentrations. Consequently, our observations may only be applicable to this subpopulation of SAH patients. Second, the relatively small number of examined samples and enrolled patients limits the extent of our conclusions. Nevertheless, the statistical relationships in our study follow the pattern of large scale studies (e.g., high correlation between admission status and treatment outcome). Third, EVD infection and CNS microbial inflammation could potentially affect the results, but our protocols specifically aim to minimise such problems. We have assumed a relationship between CSF and interstitial fluid, but this may itself be corrupted by the pathology. Future studies should include more good-grade patients without severe complications (e.g., acute HCP) with CSF drawn by LP.

## Conclusion

Aneurysmal subarachnoid haemorrhage leads to a generalised increase of amino acids and related compounds in CSF. The patterns of concentrations differ between good and poor outcome patients. Increased EAAs are strongly indicative of poor outcome.

## Ethics Statement

This is a prospective observational study conducted in a single medical centre in accordance with the Declaration of Helsinki. The local bioethics committee approved the study protocol, consenting protocol, and consent forms.

## Author Contributions

BS conceived and designed the study. BS, NW, RJa, and RJu analysed and interpreted the patient clinical data. BU, SP, AK, and ZK performed the amino acid assay and interpreted the laboratory data. BW performed statistical analysis. BS, NW, BW, and BU wrote the manuscript.

## Conflict of Interest Statement

The authors declare that the research was conducted in the absence of any commercial or financial relationships that could be construed as a potential conflict of interest.
