# Corrigendum: Amino Acids in Cerebrospinal Fluid of Patients With Aneurysmal Subarachnoid Haemorrhage: An Observational Study

**DOI:** 10.3389/fneur.2018.00416

**Published:** 2018-06-13

**Authors:** Bartosz Sokół, Bartosz Urbaniak, Norbert Wąsik, Szymon Plewa, Agnieszka Klupczyńska, Roman Jankowski, Barbara Więckowska, Robert Juszkat, Zenon Kokot

**Affiliations:** ^1^Department of Neurosurgery, Poznan University of Medical Sciences, Poznan, Poland; ^2^Faculty of Pharmacy, Department of Inorganic and Analytical Chemistry, Poznan University of Medical Sciences, Poznan, Poland; ^3^Department of Computer Science and Statistics, Poznan University of Medical Sciences, Poznan, Poland; ^4^Department of General and Interventional Radiology, Poznan University of Medical Sciences, Poznan, Poland

**Keywords:** subarachnoid haemorrhage, amino acids, early brain injury, delayed cerebral ischaemia, biomarkers

In the original article, there were mistakes in Table 2, Table 3, Table 4, Figure 1, Figure 2, Figure 3 and Figure 4.

In all mentioned tables and figures, quantity of amino acids was described in mM instead of μM. The corrected tables and figures appear below. The authors apologize for this error and state that this does not change the scientific conclusions of the article in any way.

The original article has been updated.

**Table 2:** Differences in CSF amino acid level at day 0-3, 5, and 10 post-SAH in healthy individuals (control group) and SAH patients (study group).

**Amino acid**	**Control group**	**Study group, day 0-3 post-SAH**	**Control group vs. Study group, day 0-3 post-SAH. *P*-value**	**Study group, day 5 post-SAH**	**Control group vs. Study group, day 5 post-SAH. *P*-value**	**Study group, day 10 post-SAH**	**Control group vs. Study group, day 10 post-SAH. *P*-value**
O-phosphoethanolamine (μM)	3.00	5.95	**<0.01**	5.15	**<0.001**	6.60	**<0.001**
Ethylamine (μM)	9.80	9.40	0.798	9.65	**<0.001**	11.10	0.292
Taurine (μM)	7.00	16.00	**<0.001**	14.30	**<0.001**	10.75	**<0.01**
Asparagine (μM)	5.70	10.35	**0.01**	19.50	**<0.001**	21.75	**<0.001**
Serine (μM)	24.20	55.70	**<0.001**	75.40	**<0.001**	83.75	**<0.001**
Glycine (μM)	8.90	38.75	**<0.001**	34.85	**<0.001**	35.20	**<0.001**
Hydroxyproline (μM)	0.60	1.00	0.164	1.35	0.658	1.35	0.289
Glutamine (μM)	407.60	520.65	**<0.01**	791.10	**<0.001**	796.30	**<0.001**
Aspartic acid (μM)	0.50	1.55	**<0.001**	0.80	0.175	0.95	0.054
Citruline (μM)	1.60	2.75	**0.011**	2.55	**<0.01**	3.30	**<0.01**
Threonine (μM)	24.80	29.05	0.079	56.10	**<0.001**	63.40	**<0.001**
Beta-Alanine (μM)	20.30	22.00	**0.040**	29.00	**<0.01**	27.30	**<0.01**
Alanine (μM)	35.00	88.05	**<0.001**	114.55	**<0.001**	122.55	**<0.001**
Glutamic acid (μM)	1.20	5.40	**<0.001**	3.00	**<0.001**	2.35	**<0.01**
Histidine (μM)	11.40	28.20	**<0.001**	43.35	**<0.001**	40.20	**<0.001**
3-Methylhistidine (μM)	0.50	1.00	**<0.01**	1.40	**<0.001**	1.05	**<0.01**
2-Aminoadipic acid (μM)	0.00	1.65	**<0.001**	2.10	**<0.001**	1.45	**<0.001**
Gamma-aminobutyric acid (μM)	0.40	0.50	0.699	0.30	**0.012**	0.20	0.051
3-Aminoisobutyric acid (μM)	0.50	0.30	0.668	0.30	0.563	0.35	0.806
2-Aminobutyric acid (μM)	3.20	6.20	**<0.01**	6.60	**<0.001**	7.30	**<0.001**
Arginine (μM)	15.70	19.25	0.062	26.60	**<0.001**	24.35	**<0.01**
Proline (μM)	0.80	11.80	**<0.001**	13.95	**<0.001**	16.15	**<0.001**
Ornithine (μM)	4.30	19.80	**<0.001**	17.95	**<0.001**	17.75	**<0.001**
Cystathionine (μM)	0.30	2.05	**<0.001**	1.05	**<0.001**	1.40	**<0.001**
Cysteine (μM)	0.40	2.05	**<0.001**	1.75	**<0.001**	1.65	**<0.001**
Lysine (μM)	22.50	53.80	**<0.001**	73.05	**<0.001**	72.40	**<0.001**
Methionine (μM)	3.10	6.20	**<0.01**	11.70	**<0.001**	13.65	**<0.001**
Valine (μM)	16.20	38.65	**<0.01**	66.20	**<0.001**	76.30	**<0.001**
Tyrosine (μM)	7.80	26.70	**<0.001**	36.20	**<0.001**	37.30	**<0.001**
Isoleucine (μM)	4.40	9.65	**0.001**	13.05	**<0.001**	15.05	**<0.001**
Leucine (μM)	11.40	29.20	**<0.001**	43.50	**<0.001**	56.80	**<0.001**
Phenylalanine (μM)	9.40	26.45	**<0.001**	43.25	**<0.001**	44.45	**<0.001**
Tryptophan (μM)	1.90	9.70	**<0.001**	13.60	**<0.001**	12.85	**<0.001**

*Median levels of the amino acids are presented in the table. P-values were calculated either by Mann–Whitney or t-Student test. Amino acids increasing significantly during SAH (on day 0–3, 5, or 10) are shown in bold*.

**Table 3:** Differences in monitored parameters at day 0-3, 5, and 10 post-SAH in patients with good (GO-SAH) and poor (PO-SAH) treatment outcome.

**Monitored parameter**	**Day 0–3 post-SAH**			**Day 5 post-SAH**			**Day 10 post-SAH**		
	**GO-SAH**	**PO-SAH**	**GO-SAH vs. PO-SAH. *P*-value**	**GO-SAH**	**PO-SAH**	**GO-SAH vs. PO-SAH. *P*-value**	**GO-SAH**	**PO-SAH**	**GO-SAH vs. PO-SAH. *P*-value**
C-reactive protein (mg/l)	125.00	130.00	0.715	50.70	115.80	**0.020**	22.40	109.70	0.139
White blood cell count (10^6^/mm^3^)	9.86	17.47	**<0.001**	10.57	15.03	**0.044**	9.66	16.84	0.445
Haemoglobin (g/dl)	12.10	13.10	0.212	11.30	10.90	0.262	10.60	11.50	0.672
Temperature (^O^C)	37.00	37.00	0.736	37.40	37.20	0.273	36.90	35.70	0.512
Fibrinogen (mg/dl)	485.00	550.00	0.879	509.00	676.00	0.257	428.00	698.00	**0.014**
O-phosphoethanolamine (μM)	4.10	6.80	0.161	4.90	7.70	0.161	5.95	8.50	0.019
Taurine (μM)	13.10	19.40	**0.038**	14.60	13.40	0.514	12.20	7.35	0.138
Asparagine (μM)	9.00	11.50	0.256	30.20	12.50	0.397	22.90	20.45	0.423
Serine (μM)	45.10	76.50	0.161	77.00	61.40	0.947	79.50	94.75	0.503
Glycine (μM)	29.60	41.00	0.182	30.00	43.00	0.204	35.20	35.65	0.671
Hydroxyproline (μM)	0.70	1.30	0.066	1.70	0.80	0.518	1.35	1.10	0.793
Ethylaminev (μM)	5.80	15.80	0.102	7.90	12.10	0.134	8.85	14.50	0.35
Glutamine (μM)	489.90	602.50	0.182	837.30	505.10	0.585	852.50	715.60	0.483
Aspartic acid (μM)	0.80	2.20	**0.038**	0.40	1.00	0.071	0.95	1.30	0.551
Citruline (μM)	1.90	5.90	**0.035**	2.50	3.10	0.396	3.30	3.30	1
Threonine (μM)	25.90	50.00	0.35	86.30	34.20	0.327	63.40	57.95	0.298
Beta-Alanine (μM)	28.30	22.00	0.07	30.00	28.60	0.497	26.85	27.85	0.893
Alanine (μM)	70.70	138.10	0.109	115.50	113.60	0.447	120.30	141.70	0.954
Glutamic acid (μM)	(μM)3.40	9.30	**0.038**	2.70	5.80	**0.024**	2.00	17.10	0.269
Histidine (μM)	18.90	38.10	0.256	63.60	23.70	0.711	43.55	37.45	0.753
3-Methylhistidine (μM)	0.70	1.50	**<0.01**	1.80	1.20	0.958	1.35	1.05	0.733
2-Aminoadipic acid (μM)	1.30	1.70	0.076	1.50	4.10	0.071	1.30	2.45	**0.033**
Gamma-aminobutyric acid (μM)	0.30	0.90	**0.043**	0.30	0.30	0.932	0.35	0.15	0.266
3-Aminoisobutyric acid (μM)	0.20	0.40	0.066	0.30	0.30	0.958	0.35	0.45	0.93
2-Aminobutyric acid	4.60	8.20	0.088	11.40	5.50	0.542	10.75	6.50	0.124
Arginine (μM)	18.50	22.30	0.182	30.90	26.00	0.525	24.35	20.45	0.21
Proline (μM)	4.50	14.80	0.204	18.70	10.90	0.672	16.15	18.40	0.759
Ornithine (μM)	7.60	27.50	**0.033**	17.30	28.30	0.09	17.35	33.05	0.552
Cystathionine (μM)	0.80	2.80	**<0.01**	1.00	1.80	0.089	1.30	1.50	0.199
Cysteine (μM)	1.60	2.30	0.141	1.50	2.20	0.243	1.95	1.40	0.329
Lysine (μM)	43.00	74.50	0.095	87.10	55.90	0.341	72.40	72.60	0.612
Methionine (μM)	4.60	12.30	0.204	16.20	7.70	0.491	13.65	13.70	0.687
Valine (μM)	31.80	62.40	0.109	102.50	33.60	0.397	87.75	72.20	0.377
Tyrosine (μM)	23.00	32.50	0.083	36.60	35.80	0.876	38.60	37.30	0.676
Isoleucine (μM)	6.00	13.00	**0.045**	17.60	11.80	0.597	15.05	13.70	0.45
Leucine (μM)	22.90	39.70	0.109	80.30	29.80	0.397	60.95	50.55	0.532
Phenylalanine (μM)	21.20	38.80	0.062	51.30	32.40	0.665	45.40	44.45	0.913
Tryptophan (μM)	8.00	12.20	0.066	13.60	13.60	0.606	12.60	13.55	0.804

*Median levels of the amino acids are presented in the table. P-values were calculated either by Mann–Whitney or t-Student test. Significant differences are shown in bold*.

**Table 4:** Amino acid level changes in time in good outcome SAH patients with (GO-SAH).

**Parameter**	**Day 0–3 post-SAH**	**Day 5 post-SAH**	**Day 10 post-SAH**	**Omnibus test *P*-value**	**Day 0–3 vs. Day 5 *P*-value *Post-hoc***	**Day 0–3 vs. Day 10 *P*-value *Post-hoc***	**Day 5 vs. Day 10 *P*-value *Post-hoc***
C-reactive protein (mg/l)	125.00	50.70	27.00	**0.049**	0.345	**0.012**	0.073
White blood cell count (10^6^/mm^3^)	9.86	10.57	9.34	0.631			
Haemoglobin (g/dl)	12.10	10.60	10.50	0.066			
Temperature (^O^C)	37.00	37.30	36.90	0.382			
Fibrinogen (mg/dl)	485.00	442.00	423.00	0.327			
O-phosphoethanolamine (μM)	6.20	5.00	5.80	0.325			
Taurine (μM)	13.90	14.60	10.80	0.867			
Asparagine (μM)	8.40	18.30	21.20	0.180			
Serine (μM)	45.10	73.80	76.10	**0.031**	**0.039**	**0.013**	0.566
Glycine (μM)	29.60	32.60	42.50	0.565			
Hydroxyproline (μM)	0.70	1.10	1.40	0.094			
Ethylaminev (μM)	5.70	10.10	9.80	**0.039**	0.078	**<0.01**	0.187
Glutamine (μM)	454.30	818.70	828.30	**0.001**	**<0.01**	**<0.001**	0.565
Aspartic acid (μM)	0.70	0.40	1.20	0.215			
Citruline (μM)	1.90	2.40	2.80	0.069			
Threonine (μM)	22.90	52.50	61.20	**0.012**	0.128	**<0.01**	**<0.01**
Beta-Alanine (μM)	28.30	26.80	26.70	0.553			
Alanine (μM)	70.70	106.20	120.30	0.124			
Glutamic acid (μM)	4.50	2.70	2.10	0.898			
Histidine (μM)	16.80	38.10	37.40	0.156			
3-Methylhistidine (μM)	0.60	1.50	0.90	0.369			
2-Aminoadipic acid (μM)	1.30	1.50	1.30	0.129			
Gamma-aminobutyric acid (μM)	0.30	0.20	0.50	0.633			
3-Aminoisobutyric acid (μM)	0.20	0.30	0.30	0.215			
2-Aminobutyric acid (μM)	4.60	11.40	11.40	**0.010**	**0.019**	**<0.01**	0.427
Arginine (μM)	18.50	26.50	24.10	**0.050**	0.073	**0.012**	0.345
Proline (μM)	3.40	11.90	15.20	0.062			
Ornithine (μM)	7.60	17.30	14.30	0.368			
Cystathionine (μM)	1.00	1.00	1.20	0.648			
Cysteine (μM)	1.60	1.70	2.10	0.368			
Lysine (μM)	43.00	83.20	70.80	**0.017**	**0.017**	**<0.01**	0.735
Methionine (μM)	4.60	10.00	11.90	**0.008**	**0.029**	**<0.01**	0.200
Valine (μM)	28.20	61.60	78.10	**0.015**	**0.017**	**<0.01**	0.683
Tyrosine (μM)	23.00	35.10	28.50	**0.020**	**0.023**	**<0.01**	0.647
Isoleucine (μM)	5.90	11.10	13.60	**0.024**	0.081	**<0.01**	0.219
Leucine (μM)	22.60	39.70	60.50	**0.024**	**0.022**	**0.013**	0.801
Phenylalanine (μM)	20.90	35.20	37.50	**0.038**	**0.040**	**0.018**	0.700
Tryptophan (μM)	8.00	11.80	11.10	0.072			

*Median levels of the amino acids are presented in the table. Omnibus statistical test (Friedman or repeated measures ANOVA) revealed significant changes in concentration of 13 amino acids (shown in bold). Next, post-hoc analysis (Conover–Iman for Friedman test and Fisher for repeated measures ANOVA) was run for those 13 amino acids to specify between what time points (day 0–3, 5, or 10 post-SAH) the differences in concentration were significant (shown in bold)*.

**Figure 1:**
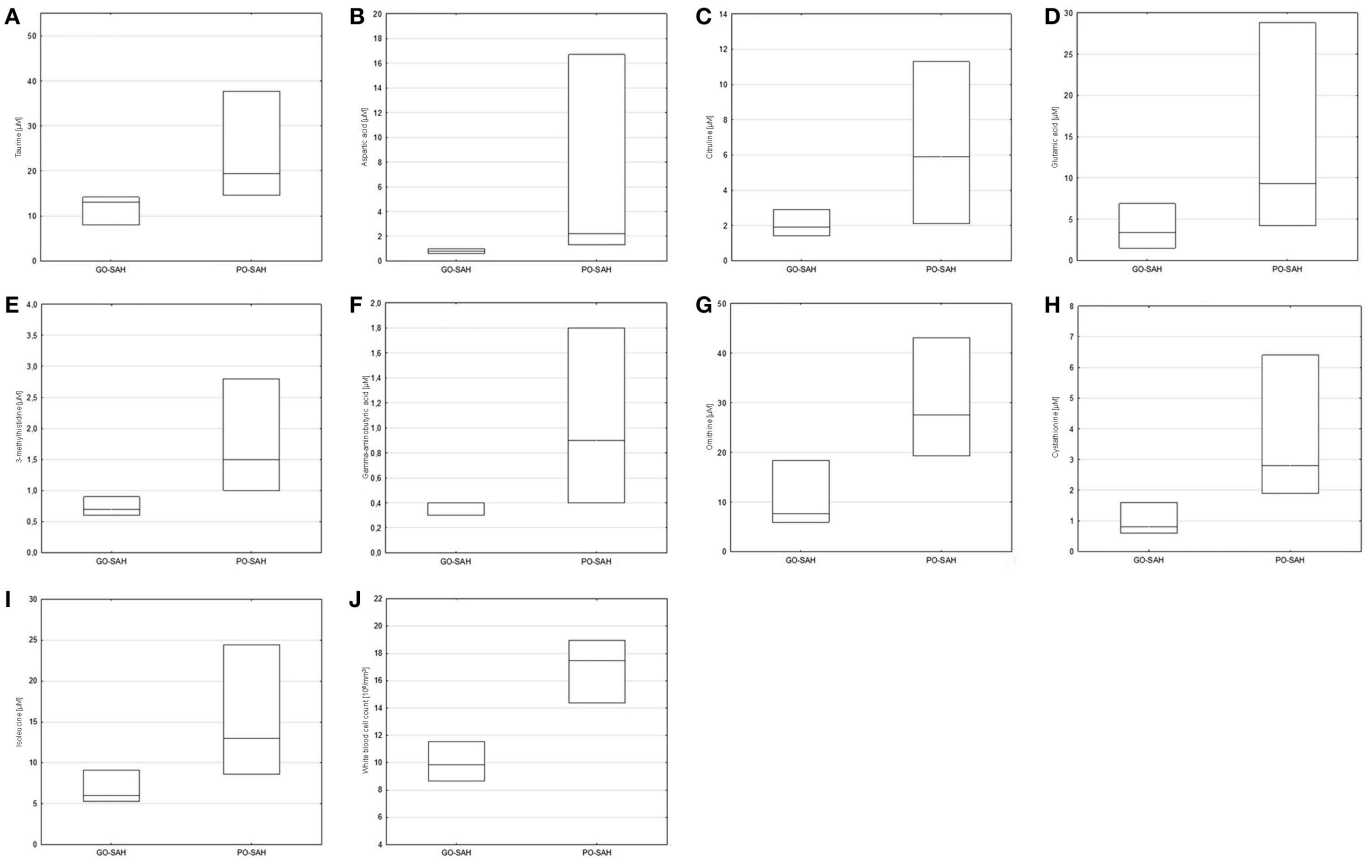
Significant differences in monitored parameters on day 0–3 post-SAH between patients with good (GO-SAH) and poor (PO-SAH) treatment outcome. Mann–Whitney test revealed significantly higher levels of: **(A)** taurine (*p* = 0.038), **(B)** aspartic acid (*p* = 0.038), **(C)** citruline (*p* = 0.035), **(D)** glutamic acid (*p* = 0.038), **(E)** 3-methylhistidine (*p* < 0.01), **(F)** gamma-aminobutyric acid (*p* = 0.043), **(G)** ornithine (*p* = 0.033), **(H)** cystathionine (*p* < 0.01), **(I)** isoleucine (*p* = 0.045) in patients with poor outcome. *T*-Student test revealed significantly higher level of white blood cell count (*p* < 0.01) in patients with poor outcome **(J)**. In all cases median levels and the 25th and 75th percentile are presented.

**Figure 2:**
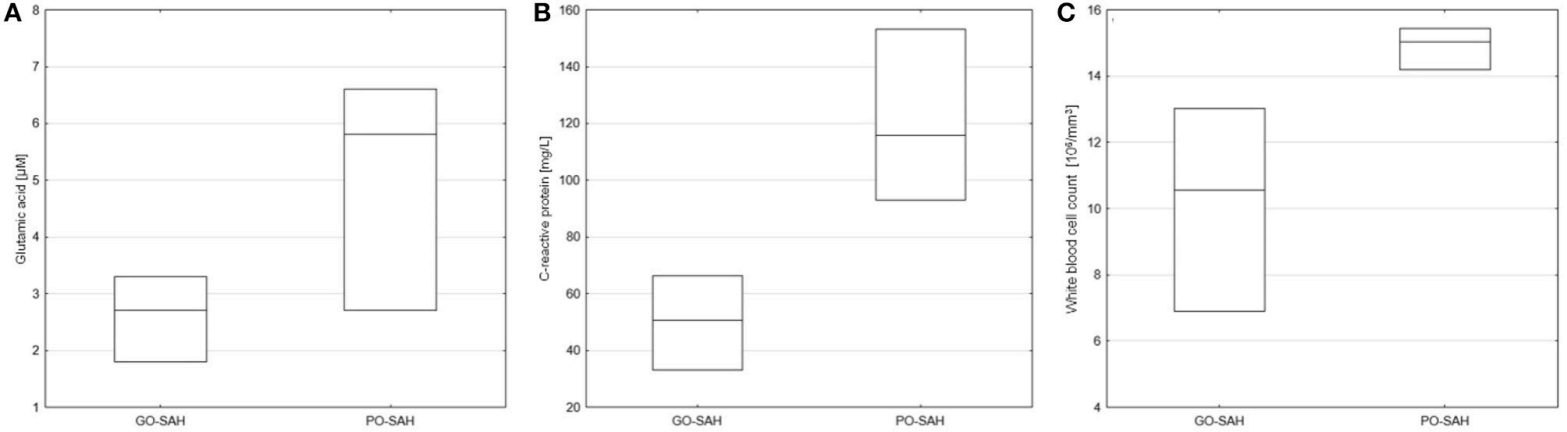
Significant differences in monitored parameters on day 5 post-SAH between patients with good (GO-SAH) and poor (PO-SAH) treatment outcome. **(A)** *T*-Student test revealed significantly higher levels of glutamic acid (*p* = 0.041) in patients with poor outcome. Mann–Whitney test revealed significantly higher C-reactive protein level (*p* = 0.020) **(B)** and white blood cell count (*p* = 0.044) **(C)** in patients with poor outcome. In all cases median levels and the 25th and 75th percentile are presented.

**Figure 3:**
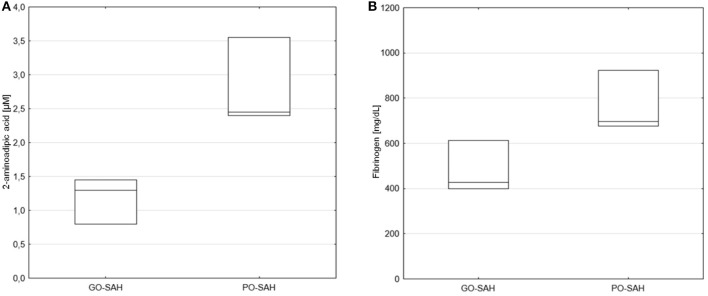
Significant differences in monitored parameters on day 10 post-SAH between patients with good (GO-SAH) and poor (PO-SAH) treatment outcome. Mann–Whitney test revealed significantly higher 2-aminoadipic acid (*p* = 0.033) **(A)** and fibrinogen (*p* = 0.014) **(B)** levels in patients with poor outcome. Median levels and the 25th and 75th percentile are presented.

**Figure 4:**
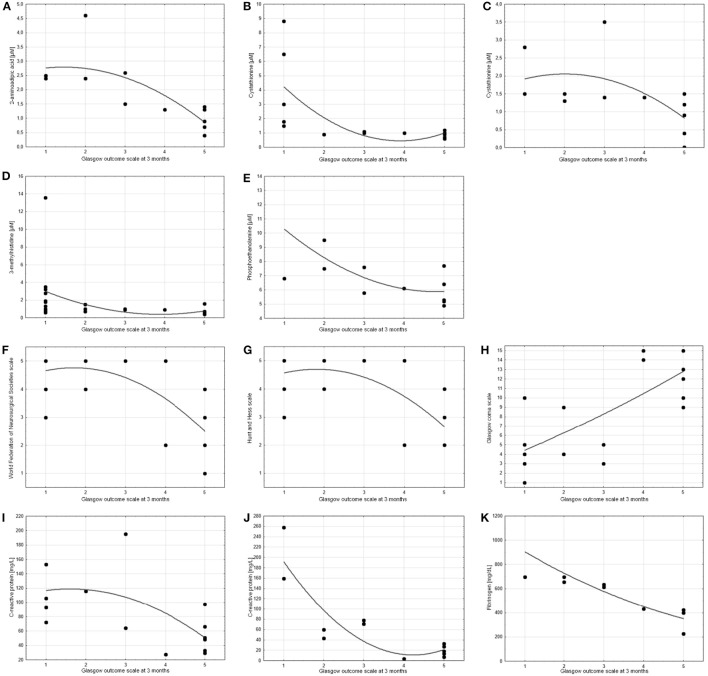
Scatter charts showing the correlation between treatment outcome (measured by Glasgow outcome scale at 3 months) and other parameters. Spearman's test revealed significant correlations (cc > 0.6 or cc < −0.6) for: **(A)** 2-aminoadipic acid on day 10 post-SAH (cc = −0.81), **(B,C)** cystathionine on day 5 post-SAH (cc = −0.72) and day 10 post-SAH (cc = −0.67), **(D)** 3-methylhistidine on day 0–3 post-SAH (cc = −0.64), **(E)** o-phosphoethanolamine on day 10 post-SAH (cc = −0.62), **(F)** World Federation of Neurosurgical Societies scale (cc = −0.64), **(G)** Hunt and Hess scale (cc = −0.61), **(H)** Glasgow coma scale (cc = 0.72), **(I,J)** C-reactive protein on day 5 post-SAH (cc = −0.64) and day 10 post-SAH (cc = −0.79), **(K)** Fibrinogen on day 10 post-SAH (cc = −0.97). *P*-values are <0.05 in all cases.

## Conflict of interest statement

The authors declare that the research was conducted in the absence of any commercial or financial relationships that could be construed as a potential conflict of interest.

